# Investigating the Use of Human Albumin in a Non-Teaching Hospital in Iran

**Published:** 2017

**Authors:** Farzaneh Foroughinia, Shahriar Mazraie

**Affiliations:** a *Department of clinical pharmacy, Shiraz University of Medical Sciences, Shiraz, Iran.*; b *Student Research Committee, Shiraz University of Medical Sciences, Shiraz, Iran.*

**Keywords:** drug utilization evaluation, albumin, non-teaching hospital, ASHP guideline, appropriate use

## Abstract

Albumin is an expensive drug which imposes relatively high cost on the health care system. Doing ABC analysis in Shahid Motahari Hospital, it was revealed that albumin is categorized in class A. Therefore, the present study aimed to evaluate the pattern of albumin use and the physiciansʹ adherence to evidenced-based albumin guidelines in this large general non-teaching hospital in Shiraz, Iran.

This study is an observational retrospective research on drug utilization. All patients admitted to Shahid Motahari hospital that had received albumin during the study period of one year (December 2013 to December 2014), were included in the study. To evaluate the appropriate use of albumin, an internal guideline was prepared using several evidence-based guidelines. Prescriptions were considered correct and appropriate if they were compliant with the standard guideline.

The result of this study indicated that about 87.3% of patients had received albumin improperly. Nephrotic syndrome without hypoalbuminemia (23.6%) was the most prevalent reason for albumin misuse and internal ward was the most consuming unit.

The findings of this study, similar to those of previous investigations in Iran, revealed the high percentage of inappropriate albumin usage in Iranian teaching and non-teaching hospitals. Regarding the high cost and short supply of this drug, educating physicians through educational programs to best implement the standard guidelines is highly recommended.

## Introduction

The development of drug utilization research (DUR) was established in 1960s when the pioneers in the field found out that the correct interpretation of information on the issue of drug usage needed rigorous research in patient levels (1). The purpose of DUR is to ensure that drugs are used in a safe, effective, and appropriate manner to improve the patientsʹ health. Pharmacists involved in DUR program can directly improve the quality of care for patientsʹ in various ways such as preventing unnecessary or inappropriate use of medication, preventing adverse reactions of drugs, and improving the overall effectiveness of the drugs as well as decreasing unnecessary medication costs (2).

To assess the quality of drug consumption, the adaptation of the use of the drugs in real cases with the worldʹs standard guidelines must be thoroughly evaluated. The quality profile may include the use of selected drugs, the medication costs, and the physiciansʹ knowledge about the drug interactions and possible side effects (1). 

Among several kinds of drugs, monitoring high cost and high usage medications are of special importance since they have the most clinical and economic impact on the drug budget. To specify such drugs, ABC analysis (the 80/20 rule) is a useful means classifying the issues into three categories: The first 20% of the drugs, accounting for approximately 80% of cumulative cost, are the A category; the second 40% of the drugs, accounting for a further 15% of cumulative cost, are the B category and the remaining 40% accounting for a mere 5% of total value, are the C category (3). Albumin is the most abundant protein in blood plasma and responsible for many important physiological functions. Two of these functions are as follows: maintenance of the colloidal osmotic pressure, and the transportation of the endogenous compounds such as fatty acids, hormones, bile acids, amino acids, metals and toxic metabolites as well as various drugs (4, 5). As to the cost, it is an expensive drug imposing a relatively high cost on the health care system. Doing ABC analysis in Shahid Motahari Hospital (a non-teaching hospital), it was revealed that albumin is categorized in class A. However, with regard to insufficient data about albumin utilization in our country, in this study, we aimed to evaluate the pattern of albumin use and also physicians´ adherence to evidenced-based albumin guidelines in a large general non-teaching hospital. To the best of our knowledge, ours is the first study in the non-teaching setting in Iran.

**Table 1:** Appropriate indications for the prescription of albumin (6).

**Indication**	**Notes**
***Appropriate indications (for which there is widespread consensus)***	
Paracentesis	5 g of albumin/L ascitic fluid removed, after paracentesis of volumes > 5 L.
Therapeutic plasmapheresis	For exchanges of > 20 mL/kg in one session or > 20 mL/kg/week in more than one session.
Spontaneous bacterial peritonitis	In association with antibiotics.
***Occasionally appropriate indications (when other criteria are fulfilled)***	
Heart surgery	Last-choice treatment after crystalloids and non-protein colloids.
Major surgery	Albumin should not be used in the immediate post-operative period. Only indication for use: serum albumin < 2 g/dL after normalisation of circulatory volume.
Cirrhosis of the liver with refractory ascites	Generally ineffective, except in patients with serum albumin < 2 g/dL.
Contraindications to the use of non-protein colloids	- pregnancy and breastfeeding; - perinatal period and early infancy; - acute liver failure; -moderate-severe renal failure (particularly when anuria/oligouria); -dialysis treatment in the presence of severe abnormalities of haemostasis and baseline albumin < 2 – 2.5 g/dL; - intracranial haemorrhage; - Hypersensitivity.
Haemorrhagic shock	Only in the case of : - lack of response to crystalloids or colloids; - Contraindication to the use of non-protein colloids.
Hepatorenal syndrome	In association with vasoconstricting drugs.
Nephrotic syndrome	Only in patients with albumin < 2 g/dL with hypovolaemia and/or pulmonary oedema.
Organ transplantation	In the post-operative period after liver transplantation to control ascites and peripheral oedema, to replace the loss of ascitic fluid from the drainage tubes, if albumin < 2.5 g/dL with a haematocrit > 30%.
Burns	In the case of burns of > 30% body surface area, after the first 24 hours.

**Table 2:** Patients’ demographic data and distribution of albumin prescription in different wards

**Variables (N=110)**	**Results**
Age (years, mean ± SD, range)	62.3±19 (7-89)
Gender: male (%)	64 (58.2)
*Admission ward, n (%)*	
Internal	83 (75.5)
CCU	3 (2.7)
Emergency	13 (11.8)
Surgery	5 (4.5)
ICU	5(4.5)
Pediatrics	1 (0.9)
*Lab value (* *mean * *± SD, range)* Serum albumin Serum creatinine *Length of hospitalization* *(Days, mean ± SD, range)*	2.8 ± 0.6 (1.7-5.1) 2.0 ± 1.4 (0.4-6.7)
Appropriate prescriptions	9.2 ± 2.7 (1-30)
Inappropriate prescriptions	11.2 ± 1.2 (1-80)

**Table 3:** Appropriate and inappropriate prescriptions of albumin by reasons of indication, total number of vials and total costs of albumin in two groups

**Indications **	**Appropriate use N (%)**	**Inappropriate use N (%)**
Cirrhosis	10 (9.1)	19 (17.3)
Hepatorenal syndrome	2 (1.8)	2 (1.8)
Nephrotic syndrome	1 (0.9)	26 (23.6)
Plasmapheresis	1 (0.9)	-
Hypoalbuminemia	-	13 (11.8)
Cerebral ischemia/brain tumor	-	7 (6.4)
Edema responsive to diuretics	-	8 (7.3)
Hemorrhagic shock responsive to crystalloids	-	3 (2.7)
Sepsis without hemodynamic shock	-	11 (10)
Acute normo-volumic hemodilution in surgery	-	7 (6.4)
Total	14 (12.7)	96 (87.3)
Total number of vials	110 (11.4)	854 (88.6)
Total cost (dollar)	5528.3 (11.4)	42920.0 (88.6)

## Methods

*Study design and population*

This study is an observational retrospective research evaluating drug utilization. All patients admitted to Shahid Motahari hospital (a general non-teaching hospital, Shiraz. Iran) who had received albumin during the one year period of the research (December 2013 to December 2014) were included. To qualify for inclusion, they must have received at least 3 doses of albumin.

*Data collection and study variables*

To collect the necessary data, a special questionnaire was designed. The required data were obtained from the pharmacy chart and the patientsʹ records. Data to be analyzed included general information about the patients, the admission wards, the patientsʹ vital signs (blood pressure), the reason(s) behind albumin prescription, blood albumin levels, consumed vials of albumin, albumin cost, duration of hospitalization, and laboratory data. 

Additional albumin costs for those who had inappropriately consumed albumin, were calculated through multiplying the total number of unnecessary vials by direct expense of one albumin vial.

*Defining criteria for appropriate use of albumin*

To evaluate the appropriate use of albumin, an internal guideline was prepared using several evidence-based guidelines (6, 7). Prescriptions were considered correct and appropriate if they were compliant with the standard guideline. Appropriate indications for the prescription of albumin are summarized in table 1.

*Data analysis*

Once collected, all data were analyzed by the SPSS software version 22. For continuous variables, mean and standard deviation were provided. The appropriate usage of albumin was reported by number and percentages of correct prescriptions with regard to the standard guideline.

## Results

In this study, a total of 110 patients were investigated, of whom 58.2% and 41.8% were male and female, respectively. The age range of patients was 7-89 years with the average of 62.3±19 years. The most distributions of albumin were observed in the internal ward with 75.5 % of total prescriptions. The patients’ demographic data and number of patients studied per ward are listed in table 2.

The average duration of hospitalization of patients who had been prescribed albumin in line with standard guideline was 9.2 ± 2.7 days and 11.2 ± 1.2 days for those who had received albumin incongruent with the guideline.

Serum albumin in patients varied from 1.7 to 5.1, with the mean level of 2.8 ± 0.6. Determining the level of serum albumin is important in that, in some cases, it is necessary to have the serum albumin level below 2gr/dl to assume the albumin administration as appropriate. In this study, only 15 patients (13.6%) had an albumin serum level below 2gr/dl.

In the present study, it was found that only 12.7% of albumin had been correctly prescribed. Among the patients having received albumin appropriately, ten had received it for cirrhosis with refractory ascites and hypoabuminemia; two for hepatorenal syndrome with concurrent usage of vasoconstrictor drugs; one for therapeutic plasmaphoresis, and the remaining for the treatment of nephrotic syndrome with concurrent hypoalbuminemia. 

On the other hand, 87.3% of patients had improperly received albumin. Nephrotic syndrome without hypoalbuminemia (23.6%) was the most prevalent reason for albumin misuse. Other inappropriate prescriptions were as follows: hypoalbuminemia in excess of albumin level of 2gr/dl, sepsis without septic shock, cirrhosis without hypoalbuminemia, edema responsive to diuretics, cerebral ischemia, chronic kidney disease, hypovolumia, and others (table 3). 

Totally, 854 albumin vials had been inappropriately administered to the patients, while 110 vials had been used appropriately. The additional expenses of albumin misuse were calculated to be about 42920 dollars (141635900 toman). This expended about 88.6% of total cost for albumin in a year studied.

## Discussion

The World Health Organization employs several intervention strategies including administrative, trainings, and monitoring actions in order to promote the rational use of medicines and to improve drug management systems. It is recognized that these strategies provide easier access to medicines in public health facilities, which economically benefit poor patients (8).

Taking the available medical budget into account, it would be possible to serve and support more patients if medicines were correctly prescribed and rationally consumed. Therefore, it is important to specify the expensive yet widely prescribed drug productions and to reach a proper consuming pattern. Although time-consuming, DUR has proved to be a useful tool to commence a debate among doctors and pharmacists in order to obtain high standards of drug usage in hospitals. It helps in revealing the pattern of drug administration in the investigated hospitals (9).

The present study indicates that 87.3% of albumin prescriptions were inappropriate. Nephrotic syndrome with the albumin serum level over 2gr/dl was the most common situation for which albumin was improperly applied. Liver cirrhosis with the albumin serum levels higher than 2gr/dl was the second reason. 

Although expensive, albumin is a widely consumed drug. In the past thirty years, several clinical studies have been conducted resulting in introducing treatment guidelines that have aimed to improve the therapeutic use of albumin. Meanwhile, Studies have shown that unfortunately about 50% to 70% of albumin administrations have been inappropriate in different health care systems with the financial impact that becomes exerted to the community (10, 11). Also, although rare, unnecessary use of human albumin may lead to allergy, complications, and side effects (12).

In one study performed in two Spanish hospitals, it was revealed that human albumin is often administered in the internal and gastrointestinal departments and that only 8.1% of the total number of vials were properly administered. It also showed that use of albumin as a first-line therapy (before using any crystalloid or other colloid) to reform the blood volume was the main reason of inappropriate administration of albumin (13).

In another study evaluating albumin prescription in a hospital in Istanbul in a 2- yearʹs period, cardiac surgery and internal wards were the departments where albumin was most frequently used. 50.4% of albumin indications proved improper in this study and the most misuse of albumin was reported to be using it as dietary supplements (33.6 %) (12). 

Another study was performed including three French hospitals. Within the two months of the study, human albumin was used most frequently for hypoalbuminemia (33%) and plasmapheresis (30.2 %) (14).

Few studies have been conducted evaluating albumin use in our country, Iran. In a study performed over 69 patients in a university affiliated hospital in Tehran, it was found that the most albumin-consuming departments were intensive care units (ICU) consuming 49.3% of total albumin prescriptions. Albumin administration was not appropriate in 36.2% of cases. Hypoalbuminemia and nutritional supplements were the most inappropriate reasons for albumin consumptions (15).

According to another study performed over 135 patients in an academic hospital in Tehran, albumin had been used mostly in volume expansion after the heart surgery (53.3%), nutrition source in malnourished patients (19.3%), paracentesis (12.9%), plasmapheresis (9.6%), hypoalbuminemia (3%) and the others (2.1%). Therefore, 67.9% of albumin vials were used inappropriately in this study (16).

Based on the reports of various studies, it seems that physicians had not been attentive enough about serum level of albumin. Thatʹs probably why, in many cases, albumin had been used despite albumin level being beyond 2gr/dl. Also, in some cases where albumin should have been used as a second-line remedy (e.g., shock), in our study and other similar studies, it had been used incorrectly as a first-line therapy. 

In this study, internal ward was the department with the most consumption. This can be due to the fact that critically ill beds are so limited in the investigated hospital (4 beds); therefore, majority of ill patients are admitted to internal ward. Additionally, our results showed that more inappropriate pattern in the usage of albumin was practices in the investigated non-teaching hospital compared to the teaching centers studied in our country. This finding is not surprising since teaching hospitals are more likely to implement standard guidelines.

The major limitation of this study was its retrograde nature which may lead to some data missing especially with regard to the precise reasons for albumin administration. Also, in few cases, albumin vials had been released from the hospital pharmacy although not administered to patients. 

## Conclusion

About 87.3% of albumin prescriptions in our hospital were not in accordance with ASHP guideline. The result of this study was consistent with those of previous investigations revealing the high percentage of inappropriate albumin usage in Iranian teaching and non-teaching hospitals. Taking the soaring cost of this drug and its short supply, it is understandable that it is necessary to alert physicians about the current trends and to educate them through educational programs about how to best implement standard guidelines in their practice. 
